# Safety, Tolerability, and Pharmacokinetics of Mevidalen (LY3154207), a Centrally Acting Dopamine D1 Receptor‐Positive Allosteric Modulator (D1PAM), in Healthy Subjects

**DOI:** 10.1002/cpdd.874

**Published:** 2020-10-07

**Authors:** Darren Wilbraham, Kevin M. Biglan, Kjell A. Svensson, Max Tsai, William Kielbasa

**Affiliations:** ^1^ Eli Lilly and Company Bracknell UK; ^2^ Eli Lilly and Company Indianapolis Indiana USA

**Keywords:** dopamine, mevidalen (LY3154207), pharmacokinetics, safety, tolerability

## Abstract

Activation of the brain dopamine D1 receptor has attracted attention because of its promising role in neuropsychiatric diseases. Although efforts to develop D1 agonists have been challenging, a positive allosteric modulator (PAM), represents an attractive approach with potential better drug‐like properties. Phase 1 single‐ascending‐dose (SAD; NCT03616795) and multiple‐ascending‐dose (MAD; NCT02562768) studies with the D1PAM mevidalen (LY3154207) were conducted with healthy subjects. There were no treatment‐related serious adverse events (AEs) in these studies. In the SAD study, 25‐200 mg administered orally showed dose‐proportional pharmacokinetics (PK) and acute dose‐related increases in systolic blood pressure (SBP) and diastolic blood pressure DBP) and pulse rate at doses ≥ 75 mg. AE related to central activation were seen at doses ≥ 75 mg. At 25 and 75 mg, central penetration of mevidalen was confirmed by measurement of mevidalen in cerebrospinal fluid. In the MAD study, once‐daily doses of mevidalen at 15‐150 mg for 14 days showed dose‐proportional PK. Acute dose‐dependent increases in SBP, DBP, and PR were observed on initial administration, but with repeated dosing the effects diminished and returned toward baseline levels. Overall, these findings support further investigation of mevidalen as a potential treatment for a range of neuropsychiatric disorders.

The catecholamine neurotransmitter dopamine plays an important role in central functions including cognition, motor activity, wakefulness, mood, and positive reinforcement.^1^, ^2^, ^3^ Dopaminergic therapeutics are available for several disorders such as dopamine agonists for Parkinson's disease, dopamine releasers for attention deficit hyperactivity disorders and narcolepsy, dopamine reuptake inhibitors for depression, and dopamine receptor antagonists for schizophrenia and bipolar disorders.^4^ Dopamine acts through binding to G‐protein‐coupled receptors, which are subdivided into 2 groups: D1‐like (D1 and D5 receptors) and D2‐like (D2, D3, and D4 receptors).^5^, ^6^

The dopamine D1 and D2 receptors are the most abundant subtypes in the brain, with at least 10‐fold higher expression than the D3, D4, and D5 receptors.^5^, ^6^ The highest expression of dopamine D1 and D2 receptors is seen in the basal forebrain, including the striatal and limbic areas, which are important for motor activity and mood/positive reinforcement.^5^ In addition, compared with the other dopamine receptor subtypes, the D1 receptor subtype shows relatively high expression in mesocortical projections to the prefrontal cortex, a brain area of key importance for higher cognitive functions including working memory, attention, and executive function.^7^, ^8^, ^9^ Impaired function of these cognitive domains remains an unmet medical need for several disorders including schizophrenia and neurodegenerative disorders such as Parkinson's and Alzheimer's disease. Based on the expression of D1 receptors in the mammalian brain, D1 agonists (also referred to as orthosteric agonists that bind to the dopamine‐binding site) have been a target of drug discovery efforts to develop improved therapies for movement disorders such as Parkinson's disease and for cognitive disorders.^7^, ^9^ In particular, the urgent need to find more effective treatments for cognitive impairment across neuropsychiatric disorders has focused around D1 receptor agonists.^10^, ^11^ However, despite strong preclinical validation support, early efforts to develop novel D1 agonists based on catechol structures have been unsuccessful. This is because of several challenges including poor metabolic stability, inverted U‐shaped dose‐response on cognition, rapid tolerance development, and in general poor tolerability of these molecules.^10^, ^11^, ^12^ More recent efforts with noncatechol‐based D1 agonists have focused on selectivity for specific downstream signaling pathways with encouraging preclinical and clinical motoric data.^13^, ^14^

An alternative approach to augment dopamine D1 function would be through allosteric potentiation, also known as positive allosteric modulation (PAM) of dopamine at the D1 receptor. We hypothesized that a selective D1PAM should enhance dopamine D1 function by increasing the affinity of dopamine when and where it is released, in contrast with a D1 agonist which will activate all D1 receptors to which it has access for as long as it is present. As a result, the D1PAM mode of action could lead to a more physiological approach with lower propensity for overstimulation (inverted U‐shaped dose response) at high doses and rapid tolerance development when compared to D1 agonists. We have recently been able to confirm this hypothesis preclinically through testing of novel tetrahydroisoquinolines, including DETQ, that show drug‐like properties with high selectivity as D1PAMs.^1^, ^15^, ^16^, ^17^, ^18^ The central nervous system (CNS) pharmacology of DETQ clearly aligns with D1 agonists but with some important differences including lack of inverted U‐shaped dose response and rapid tolerance development.^1^ Also, in vitro studies using receptor chimeras and site directed mutagenesis identified a novel dopamine D1 intracellular allosteric binding site where binding of tetrahydroisoquinoline D1PAMs results in enhanced affinity of dopamine to the orthosteric site at the human D1 receptor.^19^ The chemical structure of mevidalen (LY3154207) has been reported previously.^18^ Mevidalen is a close structural analogue of DETQ, is a centrally acting and potent D1PAM (EC_50_ of 3 nM in the human D1 cAMP assay) with specificity and high selectivity for the human D1 receptor (>1000‐fold vs other targets).^18^ In vivo testing revealed a favorable behavioral profile similar to that of DETQ with enhanced release of cortical acetylcholine and reversal of hypomotility after partial dopamine depletion. Data from nonclinical studies indicate that mevidalen is orally bioavailable and extensively metabolized. Also, mevidalen has been shown to cross the blood‐brain barrier.^18^

To our knowledge, there have been no published reports on clinical evaluation of a D1PAM. For the first time we describe here the results of safety, tolerability, and PK evaluations from a phase 1 single‐ascending‐dose (SAD) study (NCT03616795) and a multiple‐ascending‐dose (MAD) study (NCT02562768) in healthy subjects with the D1PAM mevidalen.^1^ Currently, mevidalen is under clinical development in phase 2 for cognition in Lewy body dementias (NCT03305809).

## Methods

The SAD and MAD studies were both performed at sites in the United States (PRA Health Sciences, Salt Lake City, Utah, and Parexel International LLC, Glendale, California, respectively). Protocols for both SAD and MAD studies were reviewed and approved by an independent institutional review board (Schulman Associates IRB, Cincinnati, Ohio, and Aspire IRB, Santee, California, respectively) before study start. Written informed consent was obtained from all subjects before study participation. Both studies were conducted in accordance with the International Council on Harmonization guideline for Good Clinical Practice and the original principles embodied by the Declaration of Helsinki. The studies are registered at ClinicalTrials.gov as NCT03616795 (SAD) and NCT02562768 (MAD).

### Subjects

Key inclusion criteria for the SAD study were overtly healthy men or women (not of childbearing potential) aged 18 to 70 years with a body mass index (BMI) of 18.0 to 35.0 kg/m^2^. Key inclusion criteria for the MAD study were healthy men or women (not of childbearing potential) at least 20 years of age, with a BMI of 18.0 to 29.9 kg/m^2^, inclusive. For the SAD and MAD studies, subjects were excluded if they had a significant abnormality in the 12‐lead electrocardiogram (ECG), hypertension, or medical history capable of significantly altering the absorption, metabolism, or elimination of drugs; believed to increase the risk associated with taking the study medication; or of interfering with the interpretation of data. Subjects with serious or active medical or ongoing psychiatric disorders, those who had used or intended to use over‐the‐counter or prescription medication including herbal medications within 14 days for the SAD or 7 days for the MAD prior to dosing were excluded.

### Study Design and Drug Administration

**Figure 1:**
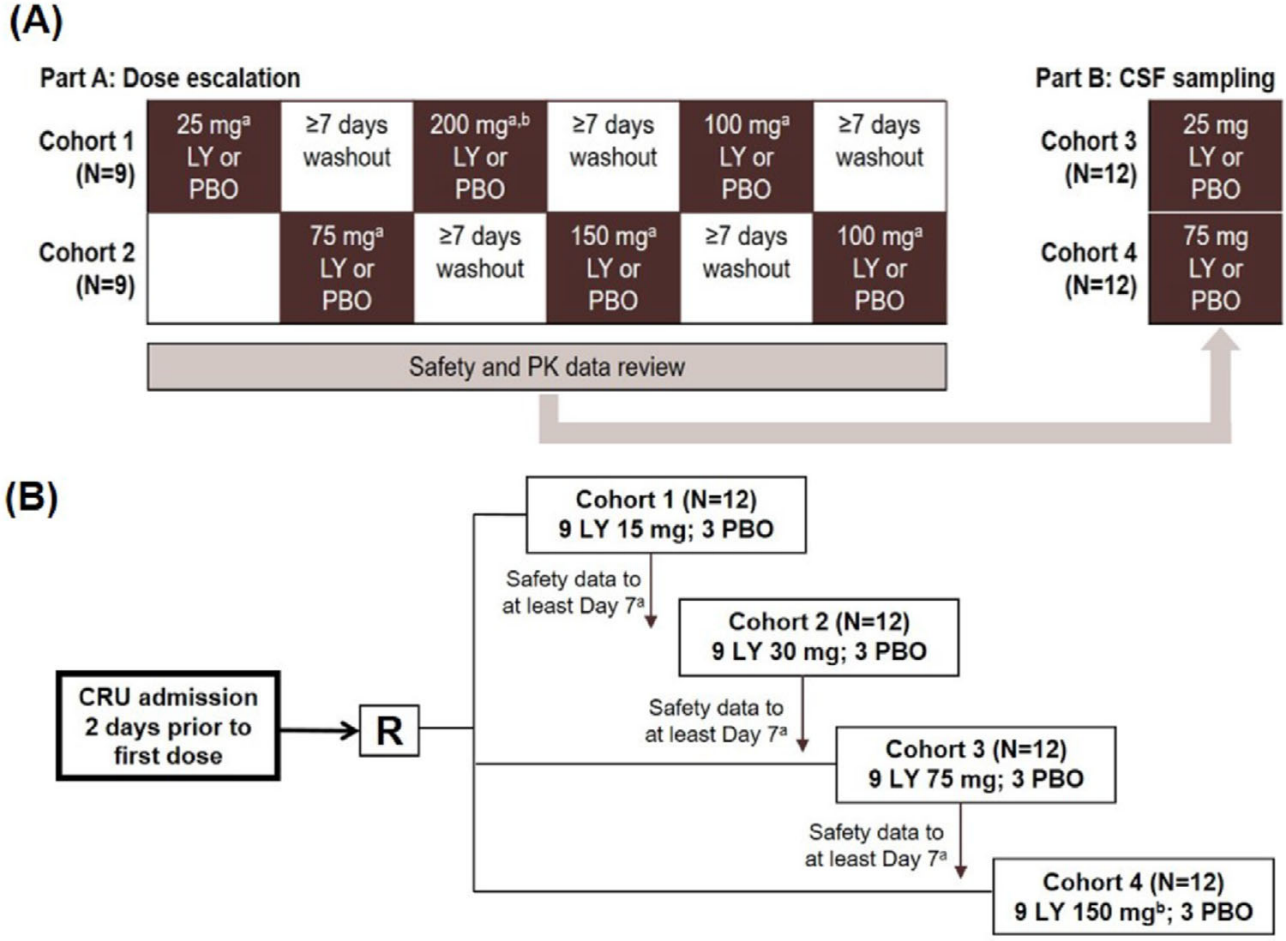
(A) Design of single ascending dose. Part A: subjects were randomized to mevidalen (n = 6) or placebo (n = 3) in each cohort in each dosing period; part B: subjects were randomized to mevidalen (n = 8) or placebo (n = 4) in each cohort. ^a^Safety review completed after each dose level prior to escalation. ^b^Dose escalation was terminated at 200 mg owing to cardiovascular effects. LY, mevidalen; PBO, placebo. (B) Design of multiple ascending dose. Each cohort contained 12 subjects (mevidalen, n = 9; placebo, n = 3) dosed daily for 14 days. ^a^Each ascending dose cohort commenced only after review of the safety data to at least day 7 from the previous cohort. ^b^The planned cohort 4 dose of 200 mg was reduced to 150 mg on the basis of emerging safety data from cohort 3. CRU, clinical research unit; CSF, cerebrospinal fluid; LY, mevidalen; PBO, placebo; R, randomized.

The SAD study was a subject‐ and investigator‐blind, randomized, and placebo‐controlled study in healthy subjects (Figure 1). The SAD study was further divided into part A and part B. Part A consisted of 2 cohorts of 9 subjects each (cohorts 1 and 2) in a 3‐period crossover design. Part B was a single‐dose, single‐period parallel design with collection of cerebrospinal fluid (CSF) in 2 cohorts of 12 subjects each (cohorts 3 and 4). Cohorts 1 and 2 (part A) were randomized to receive 2 single oral doses of mevidalen hydroxybenzoate (25, 75, 100, 150, and 200 mg) and 1 oral dose of placebo over 3 study periods. For each study period, 6 subjects received mevidalen and 3 subjects received placebo, with a washout of at least 7 days between periods. Dose escalation occurred after review of safety data from the preceding dose level. Subjects in cohorts 3 and 4 (part B) were randomized to mevidalen (8 subjects per cohort) or placebo (4 subjects per cohort), resulting in 8 subjects randomized to each treatment: 2 dose levels of mevidalen (25 and 75 mg) and placebo. In parts A and B of the study, after an overnight fast of at least 8 hours, mevidalen or placebo was administered orally in a sitting position. Subjects were fasted for at least 4 hours after dosing. Subjects were not allowed to lie supine for 2 hours after dosing, unless clinically indicated or for study procedures. Procedures in part B were similar to those in part A, but in part B a lumbar catheter was inserted prior to dosing on day 1 to allow serial CSF sampling.

The MAD study was a randomized, subject‐ and investigator‐blind, placebo‐controlled, parallel‐arm study to evaluate the PK, tolerability, and safety of once‐daily oral doses of mevidalen (15, 30, 75, or 150 mg) given for 14 days in healthy subjects (n = 48). This study was conducted in cohorts (cohorts 1 to 4), each with 9 subjects assigned to mevidalen (up to 150 mg) and 3 subjects to placebo (Figure 1). Mevidalen or placebo was given each morning under fasted conditions following an overnight fast of at least 8 hours. Subjects were required to fast for an additional 4 hours following dosing on intensive PK sampling days. Subjects were admitted to the clinical research unit 2 days prior to the first dose (day −2) and were required to remain in‐house for the whole study period until at least 24 hours after the final dose of study drug.

### Pharmacokinetic Assessments and Analyses

In part A of the SAD study, blood was collected predose and 0.5, 1, 2, 3, 4, 6, 8, 12, 16, 24, and 48 hours postdose to measure plasma concentrations of mevidalen. Urine was collected from 0 to 24 hours postdose to measure urinary concentrations of mevidalen for the characterization of renal clearance. The plasma PK parameters were maximum plasma concentration (C_max_), time to maximum concentration (t_max_), AUC from time 0 to infinity (AUC_0‐∞_), and terminal elimination half‐life (t_1/2_). In part B of the SAD study, blood was collected predose and 0.5, 1, 2, 3, 4, 6, 8, 12, 16, 24, 30, 36, and 48 hours postdose to measure plasma concentrations of mevidalen. Also, CSF was collected predose, and 1, 2, 3, 4, 6, 8, 12, 16, and 24 hours postdose to measure concentrations of mevidalen.

In the MAD study, blood was collected predose, and 0.5, 1, 2, 3, 4, 5, 6, 8, 12, and 24 hours postdose on days 1, 7, and 14 to measure plasma concentrations of mevidalen. Urine was collected from 0 to 24 hours postdose on day −1 (ie, before dosing) and on days 1, 7, and 14 to measure urinary concentrations of mevidalen for the characterization of renal clearance. The primary parameters for analysis were C_max_, t_max_, and area under the concentration‐versus‐time curve during the dosing interval (AUC_0‐tau_). Further details on sample preparation and analysis are provided in the Supplemental Information.

### Safety Assessments and Analyses

Safety data included documentation of adverse events (AEs), safety laboratory parameters, ECG parameters, Columbia suicide severity rating scale (MAD only), vital signs, and ambulatory blood pressure monitoring (ABPM). Treatment‐emergent AEs (TEAEs) were defined as events that began on or after the date of study drug administration and up to 30 days thereafter. Clinical and laboratory AEs were coded according to the Medical Dictionary for Regulatory Activities (version 17.1).

### Statistical Analyses

Pharmacokinetic parameter estimates for mevidalen were calculated using standard noncompartmental methods of analysis. In the SAD study, PK parameter estimates were evaluated to delineate effects of dose proportionality. The ratio of dose‐normalized, geometric mean values based on the power model outlined by Gough was used to assess dose proportionality.^19^ The assessment of dose proportionality was conducted based on AUC_0‐∞_ and C_max_, fitting log PK parameter against log dose and subject as a random effect. Predicted ratios of dose‐normalized means, starting with highest dose/lowest dose tested, and corresponding 90% confidence interval (CI) were estimated from the model.

For the MAD study, the plasma PK parameters C_max_ and AUC_0‐tau_ obtained for mevidalen were used to estimate the dose‐exposure proportionality of mevidalen using a linear mixed‐effects power model. Both C_max_ and AUC_0‐tau_ were log‐transformed prior to analysis, and the log‐transformed dose was the independent variable, and subject was a random effect. The least‐squares (LS) means for each treatment, together with the treatment differences and associated 90%CIs, were estimated and repeated for days 1, 7, and 14. For each ABPM parameter, the change from baseline was analyzed using a mixed‐model repeated‐measures analysis, and the LS means and 90%CIs were reported.

## Results

### Subject Disposition and Baseline Characteristics

In the SAD study, overall, 42 subjects (38 men, 4 women) aged between 18 and 59 years were randomized in the study and received at least 1 dose of study drug with a total of 18 subjects (16 men, 2 women) in part A and a total of 24 healthy subjects (22 men, 2 women) in part B. Subject demographics are presented in Table S1. Most subjects randomized in this study were white (89% in part A and 92% in part B). Of the 18 subjects who entered part A of the study, 17 completed the study (2 doses of mevidalen and 1 dose of placebo); see Table S1A. One subject (subject 0208) was withdrawn because of an ongoing AE of anxiety following 150 mg mevidalen in period 2 and did not receive the scheduled dose of 100 mg mevidalen in period 3. All 24 subjects who entered part B of the study received a single dose of mevidalen or placebo and completed the study (Table S1B.).

In the MAD study, a total of 48 healthy subjects (43 men and 5 women) between the ages of 22 and 75 years participated (Table S2). Of the 48 subjects who were randomized, 47 completed the study, and 1 subject in cohort 4 withdrew from the study because of toothache after receiving placebo for 12 days.

### Pharmacokinetics

**Figure 2:**
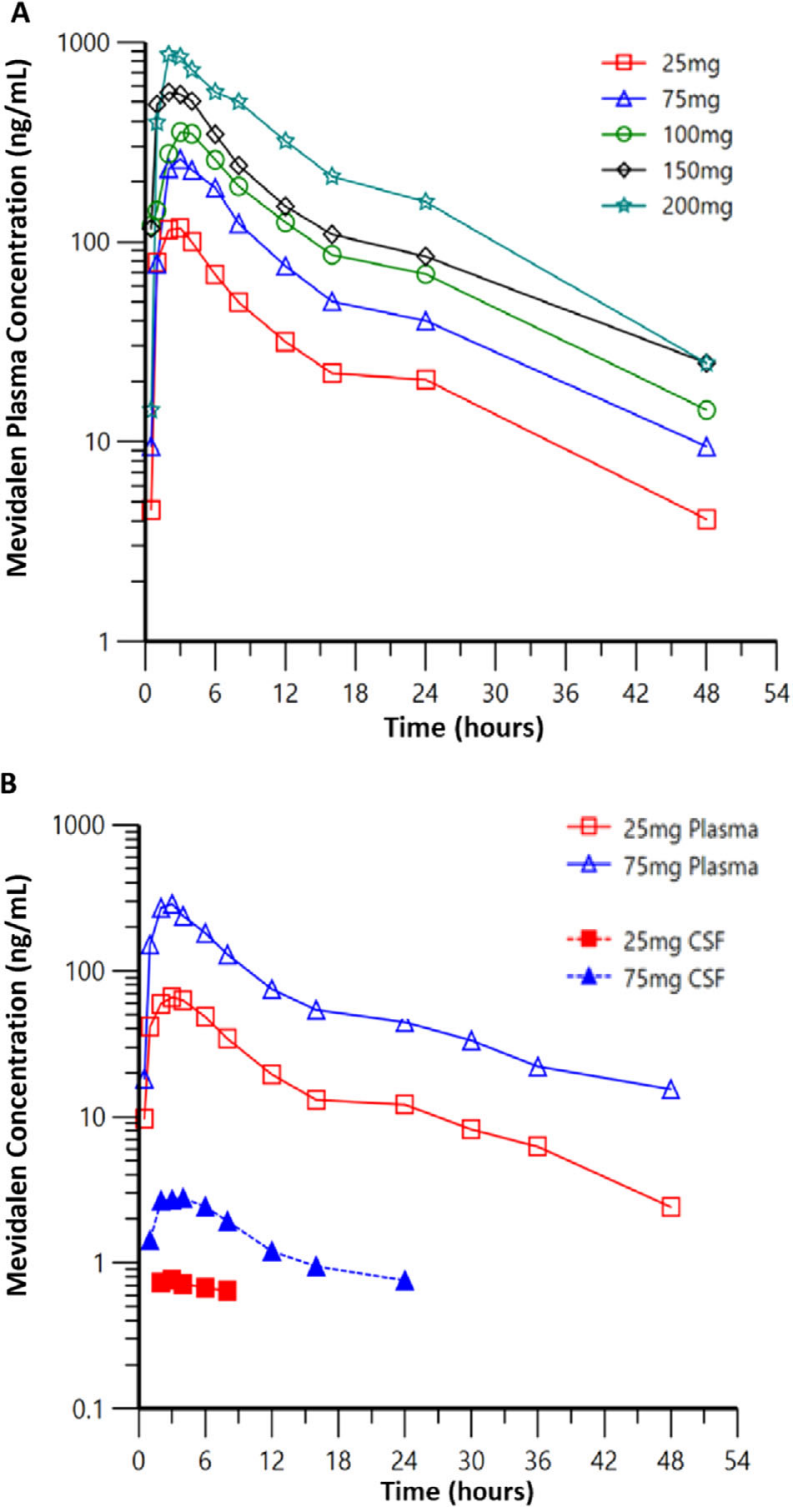
(A) Mean plasma concentration‐time profiles following single doses of mevidalen from part A of the SAD study. (B) Mean plasma and CSF concentration‐time profiles following single doses of mevidalen from part B of the SAD study.

Figure 2 illustrates the mean plasma concentration‐time profiles following single doses of mevidalen from part A of the SAD study (Figure 2A), and mean plasma and CSF concentration‐time profiles following single doses of mevidalen from part B of the SAD study (Figure 2B). Following oral administration of a single dose of mevidalen, the median t_max_ was about 2 to 3 hours, and the mean t_1/2_ was about 12 hours across doses of 25 to 200 mg (Table 1). Across the doses evaluated, the renal clearance of mevidalen ranged from about 4 to 6 mL/h, and the fraction of the administered mevidalen dose excreted in urine (Fe) was about 0.02%, indicating minimal excretion of mevidalen by the kidney (Table 1). An analysis of dose proportionality from 25 to 200 mg indicated an approximate increase of 2 times in C_max_ and AUC_0‐∞_ per doubling of the dose. The ratio of dose‐normalized means and the 90%CI were 0.90 (0.72‐1.12) for C_max_ and 1.05 (0.87‐1.27) for AUC_0‐∞_. Within‐subject variability was about 22% for C_max_ and 18% for AUC_0‐∞_. Mean mevidalen concentration‐time profile in CSF generally paralleled that of plasma following single‐dose administration of 25 and 75 mg (Figure 2B). The fraction of the mevidalen exposure in CSF relative to total plasma exposure was about 0.01 (Table 1). Furthermore, the CSF to unbound plasma exposure was about 0.3.

**Table 1:** Pharmacokinetic Parameters of Mevidalen in the SAD Study

SAD Study Part A					
Dose	25 mg	75 mg	100 mg	150 mg	200 mg
N	6	6	7	9	6
C_max_, ng/mL	130 (21)	313 (79)	416 (182)	680 (192)	937 (229)
t_max_, h^a^	2.00 (1.00–3.00)	3.00 (2.00–6.05)	3.00 (1.00–6.00)	2.00 (1.00–4.00)	2.52 (2.00–6.00)
AUC_0‐∞_, ng·h/mL	1380 (404))	3080 (802)	4750 (1960)	7000 (2790)	10800 (2800)
AUC, ng·h/mL	1060 (296)	2380 (491)	3640 (1350)	5240 (1890)	8810 (2450)
t_1/2_, h	12.8 (3.37)	13.0 (3.55)	12.0 (2.30)	14.0 (4.42)	10.7 (2.30)
Fe, %	0.0163 (0.005)	0.0242 (0.019)	0.0169 (0.006)	0.0218 (0.013)	0.0291 (0.021)
CLr, L/h	0.004 (0.001)	0.007 (0.004)	0.005 (0.002)	0.007 (0.005)	0.007 (0.005)

SAD Study Part B				
	25 mg		75 mg	
Dose	Plasma	CSF	Plasma	CSF
n	8	8	8	8
C_max_, ng/mL	78.39 (10.6)	0.83 (0.122)	315.20 (102)	3.11 (0.777)
t_max_, h^a^	2.00 (1.00‐4.00)	3.55 (2.10‐8.03)	2.00 (1.00‐3.00)	3.08 (2.07‐6.08)
t_1/2_, h	12.0 (3.27)	NC	15.2 (3.96)	9.87 (6.57)
AUC_0‐24_, ng·h/mL	637 (138)	NC	2570 (959)	32.6 (11.4)
AUC_0‐24_, CSF:plasma ratio	NC		0.0129 (0.001)	
C_max_, CSF:plasma ratio	0.0108 (0.002)		0.0101 (0.002)	

AUC_0‐24_, area under the concentration‐versus‐time curve from 0 to 24 hours; AUC_0‐∞_, area under the concentration‐versus‐time curve from 0 to infinite time; CLr, renal clearance; C_max_, maximum plasma concentration; CSF, cerebrospinal fluid; Fe, fraction of dose excreted as unchanged drug expressed as a percentage; n, number of subjects; NC, not calculated; SAD, single‐ascending dose; t_1/2_, terminal half‐life. t_max_, time of C_max_.

Data are shown as arithmetic mean and standard deviation unless noted otherwise.

^a^ Median (range).

**Figure 3:**
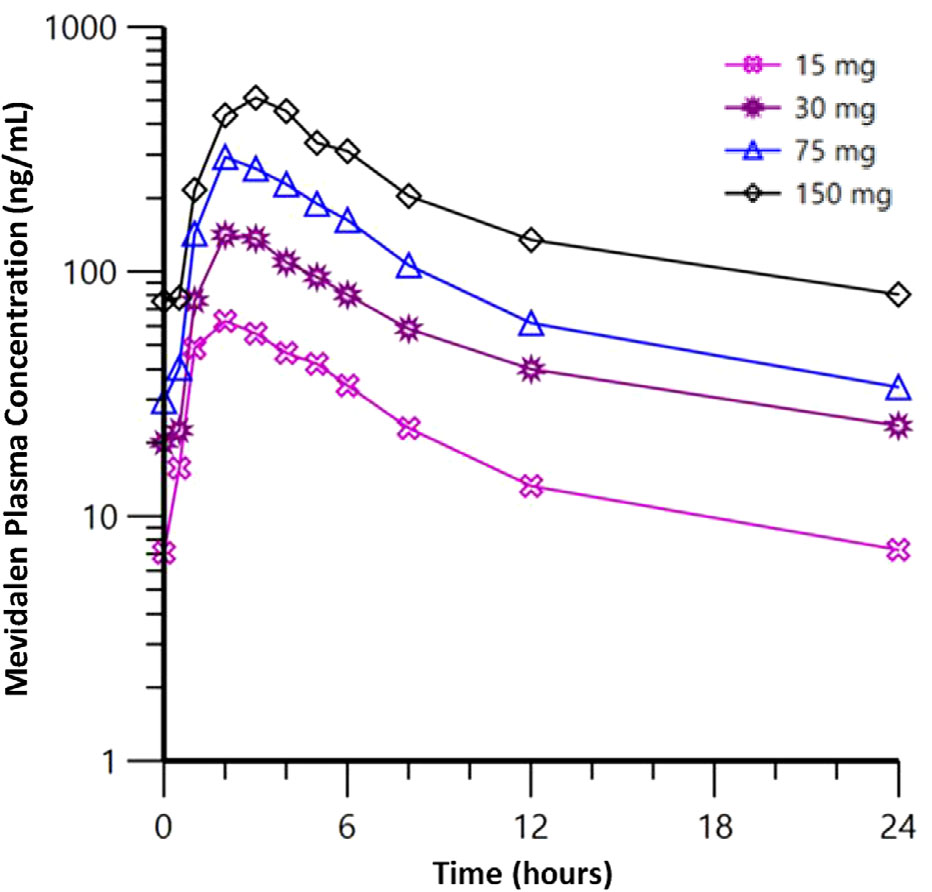
Mean plasma concentration‐time profiles on day 14 following once‐daily dosing of mevidalen.

Figure 3 illustrates the mean plasma concentration‐time profiles on day 14 following 14 days of once‐daily dosing of mevidalen. Overall, the multiple‐dose PK was consistent with single‐dose PK. On days 7 and 14, modest accumulation (R_A_) of mevidalen up to 1.34 was observed at the 15‐mg dose level; otherwise, little to no accumulation occurred at higher doses. Exposure to mevidalen increased in a dose‐related manner, with C_max_ and AUC_0‐tau_ increased by 1.82‐ and 1.87‐fold, respectively, per doubling of the dose. The ratio of dose‐normalized means (90%CIs) were 0.90 (0.70‐1.16) for C_max_ and 1.12 (0.89‐1.40) for AUC_0‐tau_ (data not shown). The rest of the PK parameters including days 1 and 7 are shown in Table 2.

**Table 2:** Pharmacokinetic Parameters of Mevidalen in the MAD Study

	Day 1				Day 7				Day 14			
Dose (mg)	15	30	75	150	15	30	75	150	15	30	75	150
n	9	9	9	9	9	9	9	9	9	9	9	9
C_max_, ng/mL	59.67 (23.1)	139.41 (49.1)	293.66 (123)	563.64 (180)	69.62 (24.4)	154.27 (43.8)	323.71 (106)	557.04 (107)	69.66 (25.2)	153.47 (37.2)	323.63 (107)	541.55 (239)
t_max_, h^a^	2.95 (0.98‐3.98)	3.00 (1.98‐6.00)	2.02 (1.97‐5.07)	3.00 (2.00‐4.95)	2.00 (1.03‐3.00)	2.00 (1.02‐5.00)	2.02 (1.02‐3.00)	2.00 (1.97‐4.00)	2.03 (0.98‐5.00)	2.02 (1.98‐3.03)	1.98 (1.93‐4.00)	3.00 (1.00‐6.02)
AUCτ, ng·h/mL	379 (97.7)	1240 (362)	2340 (736)	4820 (1240)	468 (124)	1290 (313)	2340 (725)	4680 (1130)	511 (146)	1300 (372)	2330 (733)	4510 (1330)
R_A_ AUCτ	—	—	—	—	1.23 (14)	1.04 (26)	0.99 (19)	0.97 (19)	1.34 (16)	1.03 (29)	0.99 (24)	0.92 (31)
Fe, %	—	—	—	—	NC	0.017 (0.009)	0.009 (0.004)	0.009 (0.003)	0.004 (0.008)	0.017 (0.008)	0.008 (0.005)	0.010 (0.004)
CLr, L/h	—	—	—	—	NC	0.004 (0.002)	0.003 (0.002)	0.003 (0.001)	0.001 (0.002)	0.004 (0.001)	0.003 (0.002)	0.004 (0.001)

AUCτ, area under the concentration‐versus‐time curve during the dosing interval; CLr, renal clearance; C_max_, maximum plasma concentration; Fe, fraction of dose excreted as unchanged drug expressed as a percentage; MAD, mingle ascending dose; n, number of subjects; NC, not calculated; R_A_, accumulation ratio day 14 or day 7: day 1; t_max_, time of C_max_.

Data are shown as arithmetic mean and standard deviation unless noted otherwise.

^a^ Median (range).

### Safety and Tolerability

In the SAD and MAD studies, no deaths or serious AEs occurred. The majority of AEs were mild in severity.

In the SAD study, of the randomized 18 subjects, 1 subject withdrew because of anxiety following a 150‐mg dose, and 17 subjects completed the study. A total of 111 TEAEs were reported, which were mostly mild in severity (101 of 111), with 10 reported as moderate. One subject was withdrawn because of an ongoing AE of anxiety following 150 mg mevidalen in period 2. There were 84 AEs reported by 17 subjects (94%) that were considered related to study treatment (6 following placebo; 78 following mevidalen), of which 81 were mild in intensity and 3 were moderate (Table 3A). The moderate AEs were anxiety (100 mg mevidalen), body aches (200 mg mevidalen), and agitation (placebo). The majority of related TEAEs (n = 69) were reported after doses ≥ 100 mg mevidalen. The most commonly reported (≥4 occurrences) TEAEs were insomnia, decreased appetite, anxiety, and dizziness (Table 3A). All 24 subjects who participated in part B of the study and received a single dose of mevidalen or placebo reported a total of 80 TEAEs (all causality), which were mild (n = 60) or moderate (n = 20) in severity. The most commonly reported (≥4 occurrences) TEAEs (all causality) were post‐lumbar puncture syndrome, procedural pain, and postprocedural discomfort. There were no apparent drug‐ or dose‐related trends in the clinical laboratory data and 12‐lead ECG parameters (with the exception of dose‐related changes in heart rate) following single doses of mevidalen.

**Table 3:** Summary of Treatment‐Emergent Adverse Events in the (A) SAD and (B) MAD Studies

SAD Study (A)						
	Placebo (n = 18)	25 mg (n = 6)	75 mg (n = 6)	100 mg (n = 8)	150 mg (n = 9)	200 mg (n = 6)
TEAE, n (%) [events]	5 (27.8) [6]	1 (16.7) [1]	4 (66.7) [8]	6 (75.0) [17]	8 (88.9) [34]	5 (83.3) [18]
Insomnia^a^	0	0	1 [1]	4 [4]	3 [3]	3 [3]
Decreased appetite^a^	1 [1]	0	1 [1]	1 [1]	3 [3]	2 [2]
Anxiety^a^	0	0	0	2 [2]	4^b^ [3]	1 [1]
Dizziness^a^	0	0	2 [2]	1 [1]	2 [2]	2 [2]

MAD Study (B)						
	Placebo (n = 12)	15 mg (n = 9)	30 mg (n = 9)	75 mg (n = 9)	150 mg (n = 9)	Total (n = 48)
Total number of all TEAEs	5	6	5	6	35	57
Subjects with any TEAEs, n (%)	5 (41.7)	4 (44.4)	5 (55.6)	3 (33.3)	8 (88.9)	25 (52.1)
Treatment‐related TEAEs in ≥3 subjects, n						
Insomnia	0	0	0	1	4	5
Dizziness	0	0	0	0	3	3
Nausea	1	0	0	0	2	3
Nervousness	0	0	0	0	3	3
Palpitations	0	0	0	0	3	3

MAD, multiple ascending dose; SAD, single ascending dose; TEAE, treatment‐emergent adverse event.

a TEAEs in ≥5 subjects.

b One subject discontinued owing to an adverse event of anxiety following 150 mg.

**Figure 4:**
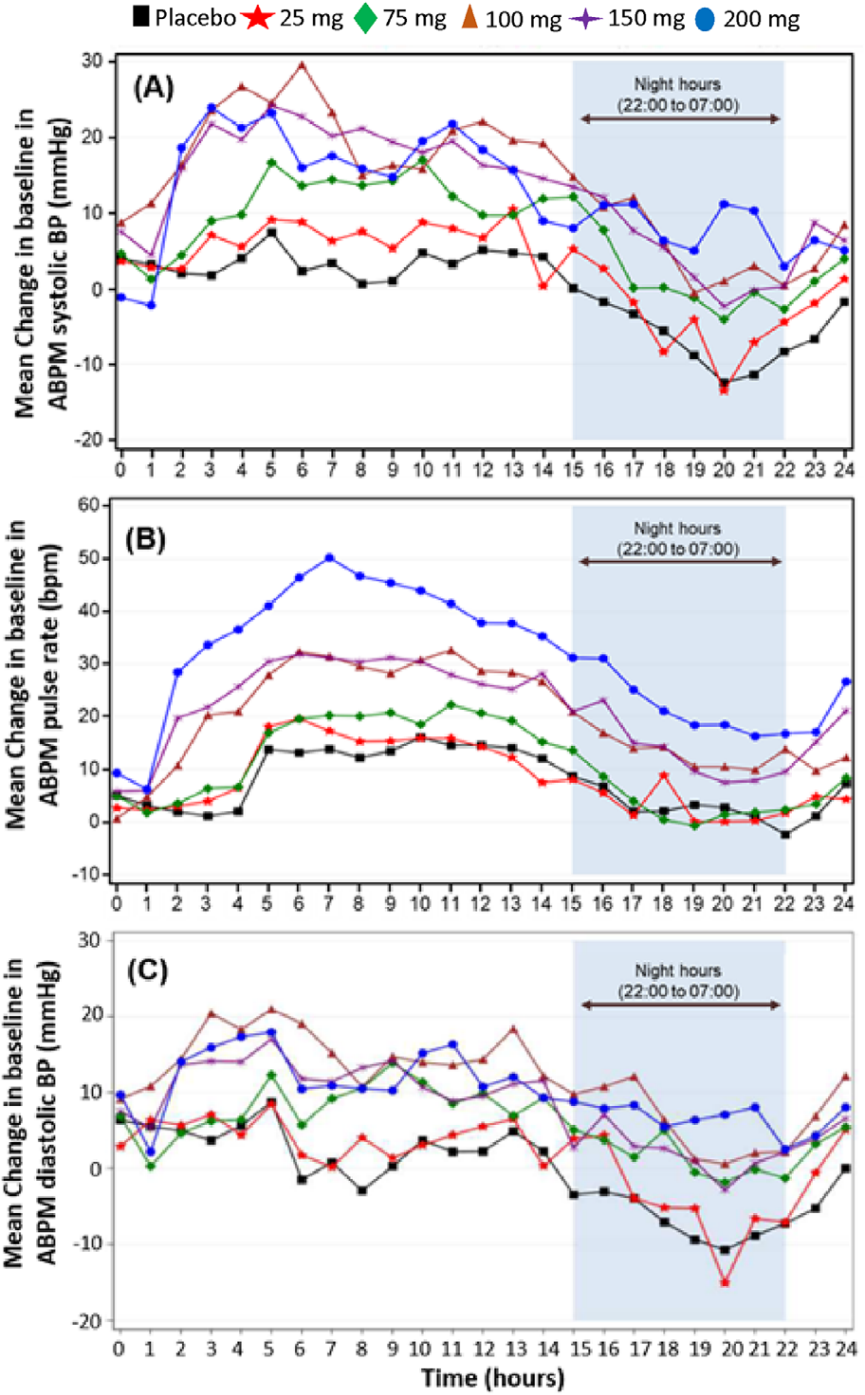
Hourly mean ambulatory blood pressure monitoring (SAD study) change from baseline in (A) systolic blood pressure, (B) pulse rate, and (C) diastolic blood pressure. ABPM, ambulatory blood pressure monitoring; BP, blood pressure.

Dose‐related increases in pulse rate (PR) and systolic blood pressure (SBP) were noted at doses ≥ 75 mg mevidalen, as well as increases in diastolic blood pressure (DBP) at doses ≥ 100 mg mevidalen that resolved by 24 hours (Figure 4). In addition, these data showed that the diurnal pattern was retained in the presence of mevidalen. The PR, SBP, and DBP showed a similar pattern over the 24‐hour postdose period; the largest effects were seen in the 4‐ to 12‐hour postdose period and decreased toward baseline thereafter.

**Figure 5:**
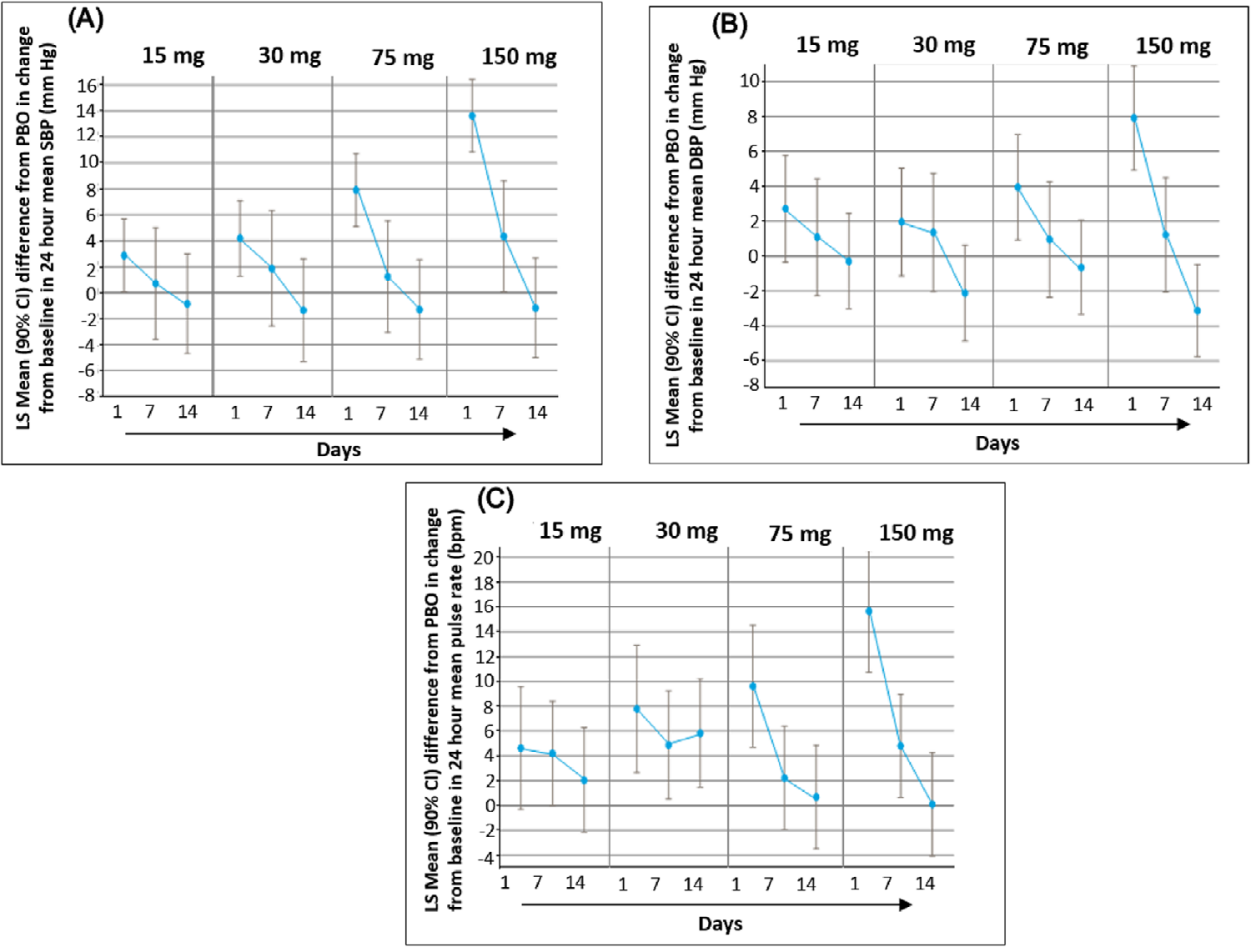
The initial dose‐related increase in (A) systolic blood pressure, (B) pulse rate, and (C) diastolic blood pressure on day 1 showed accommodation following once‐daily dosing of mevidalen. Data shown as least‐squares mean and 90% confidence interval.

In the MAD study, of the 48 subjects who received daily doses of mevidalen or placebo, 25 reported a total of 57 TEAEs (all causality; Table 3B). Common treatment‐related AEs (≥4 occurrences, primarily at the 150‐mg dose) were mild in severity and included insomnia, dizziness, nausea, nervousness, and palpitations (Table 3B). A majority of the AEs were transient and observed during the first week of dosing. There were no apparent drug‐ or dose‐related trends in the clinical laboratory data and 12‐lead ECG parameters. Dose‐related increases in mean ABPM‐derived SBP, DBP, and PR were noted on day 1, with peak increases generally between 4 and 8 hours postdose. By day 14 (day 7 for lower mevidalen doses), the magnitude of these increases was lower than that observed on day 1 and within the range observed with placebo, suggesting accommodation of these cardiovascular effects (Figure 5). Dose‐related increase (as measured by change from baseline) was observed in supine vital signs (heart rate and blood pressure [BP] parameters) similar to cardiovascular effects seen with ABPM.

## Discussion

Overall, mevidalen demonstrated an acceptable safety, tolerability, and PK profile in healthy subjects to support further development in neuropsychiatric disorders. Dose‐dependent increases in activating AEs and acute effects on BP and PR suggest a central pharmacodynamic (PD) effect consistent with D1 activation. The vital sign effects appear to accommodate after repeat dosing and should not limit clinical development.

Central dopamine D1 receptors play an important role to maintain motor activity, wakefulness, cognition, and reward/motivation.^1^, ^20^ In the SAD and MAD studies, dose‐dependent activating AEs such as anxiety, nervousness, and insomnia were observed at doses of 75 mg and are consistent with central D1 receptor activation (Table 3). This is supported by preclinical data on mevidalen^18^ and the structurally related compound DETQ.^15^, ^16^ These studies show that D1PAMs when tested in humanized D1 (hD1) mice show increased locomotor activity and wakefulness after doses that resulted in unbound brain concentrations around or slightly above the D1PAM EC_50_ value for potentiation of dopamine in the hD1 cAMP assay; D1PAM EC_50_, 2.3 nM for mevidalen.^18^ The human CSF PK study revealed that about 1% of the total exposure in plasma was available in CSF, with the CSF C_max_ 3 ng/mL (6.7 nM) at 75 mg and 0.8 ng/mL (1.7 nM) at 25 mg. The CSF C_max_ at 25 mg was slightly below the D1PAM EC_50_, with no CNS activating AE reported at this dose. However, after 75 mg the CSF C_max_ was about 3 times higher than the D1PAM EC_50_ with CNS activating AEs evident. The K_pu,u_ (unbound CSF drug vs plasma ratio) was estimated to be approximately 0.3. As discussed in Hao et al, 2019, the explanation for a K_pu,u_ of less than 1 is likely related to physicochemical properties as we have no evidence that mevidalen is a PgP substrate.^18^ Importantly, mevidalen concentrations accounting for K_pu,u_ at doses evaluated in these clinical studies are at levels expected to engage the central D1 receptor based on preclinical findings. Similar to D1 agonists, D1PAMs increase the release of both acetylcholine and histamine in the brain, as shown by in vivo microdialysis studies in the humanized D1 mouse.^16^, ^17^, ^18^ These neurotransmitters are known to play a prominent role in cortical activation and maintenance of wakefulness.^21^, ^22^, ^23^ Thus, secondary effects mediated by acetylcholine and histamine may contribute to the central pharmacology of mevidalen. Overall the human data support engagement of central dopamine D1 receptors and are consistent with the preclinical PK/PD relationship.

Dopamine has a complex role in the regulation hemodynamics both through central and peripheral mechanisms.^24^, ^25^, ^26^ Activation of renal of D1 receptors with D1 agonists including the peripherally restricted compound fenoldopam leads to decreased BP. This is the result of enhanced renal blood flow and inhibition of sodium transport, natriuresis.^27^, ^28^, ^29^ In the current study, mevidalen was found to increase SBP, DBP, and PR in a dose‐dependent fashion. These sympathomimetic effects are most likely centrally mediated and linked to the compound's potentiation of newly released neuronal dopamine in the forebrain and brain stem areas. Based on preclinical studies, key dopamine neuronal projection areas such as the striatal complex have basal extracellular dopamine levels around 50 nM,^30^ whereas peripheral tissue and circulating dopamine levels are much lower.^31^, ^32^ This could possibly explain why the main PD effects of mevidalen are centrally mediated, although further studies are warranted.

Repeated dosing with mevidalen resulted in accommodation of the increases seen in SBP, DBP, and PR at all doses tested. Depending on the dose, the time course for the accommodation varied from 7 to 14 days. This suggests that an uptitration strategy with dose increases every 7‐14 days may be useful to ameliorate this cardiovascular effect; however, this strategy has not yet been evaluated. Although the underlying mechanism is not understood, enhanced vagal response (parasympathetic activation) is a possibility to compensate for the sympathetic activation.^33^ A gradual accommodation to some of the central AEs (dizziness, insomnia, etc.) can also play a role in the hemodynamic response over time. However, in contrast to many D1 agonists, preclinical studies in the hD1 mouse with D1PAMs do not provide evidence of tolerance development to the behavioral or neurochemical effects after repeated dosing.^15^, ^17^, ^18^

Other sympathomimetic drugs including methylphenidate increase the release of both dopamine and norepinephrine with subsequent activation of all dopamine and adrenergic (alpha and beta) receptor subtypes. The alpha‐adrenergic receptors play a key role in vasoconstriction with subsequent increase in both BP and PR.^33^ In this respect, there is a clear difference in the mechanism of action compared with the selective D1PAMs, which do not enhance norepinephrine or dopamine release based on preclinical data (unpublished observations). In addition, sustained cardiovascular effects have been reported for methylphenidate and similar compounds after long‐term use.^34^ Additional chronic studies with therapeutic doses of mevidalen will help to clarify the hemodynamic profile of this new class of compounds.

## Conclusions

Mevidalen demonstrated an acceptable safety, tolerability, and PK profile in healthy subjects to support further development in neuropsychiatric disorders. Acute increases in vital signs and activating AEs may limit the clinical utility of mevidalen at doses greater than 75 mg. Mevidalen is currently in phase 2 development for the symptomatic treatment of cognition in Lewy body dementias (NCT03305809). Doses were selected based on targeting mevidalen exposures that demonstrated pharmacological activity in preclinical experiments, and the desire to explore the effect of different doses on relevant clinical domains (cognition, motor, and wakefulness) and cardiovascular effects.

## Conflicts of Interest

Darren Wilbraham, Kevin M. Biglan, Kjell A. Svensson, Max Tsai, and William Kielbasa are employees of Eli Lilly and Company Inc., and may own stock in this company.

## Funding

This study was funded by Eli Lilly and Company Inc.

## Supporting information

Supplementary information

Supplementary information

Supplementary information

Supplementary information
