# Supplementary material for: Safety, Tolerability, and Pharmacokinetics of Mevidalen (LY3154207), a Centrally Acting Dopamine D1 Receptor‐Positive Allosteric Modulator (D1PAM), in Healthy Subjects
Source: Clin Pharmacol Drug Dev. 2020 Oct 7;10(4):393–403. doi: 10.1002/cpdd.874 (PMC8048550; doi:10.1002/cpdd.874)
Supplement: Supplementary file 1 — Supplementary information [file CPDD-10-393-s004.doc]

**Table S1A.** **SAD-Subject Demographics and Other Baseline Characteristics**

| **Part A** | | | | |
| --- | --- | --- | --- | --- |
|  |  | Cohort 1 | Cohort 2 | Overall |
| **Number of Subjects** |  | 9 | 9 | 18 |
| **Age (Years)** | Mean (SD) | 31.0 (8.8) | 36.2 (16.3) | 33.6 (13.0) |
| **Sex** | Male | 9 (100.0%) | 7 ( 77.8%) | 16 ( 88.9%) |
|  | Female | 0 ( 0.0%) | 2 ( 22.2%) | 2 ( 11.1%) |
| **Ethnicity** | Hispanic or Latino | 3 ( 33.3%) | 3 ( 33.3%) | 6 ( 33.3%) |
|  | Not Hispanic or Latino | 6 ( 66.7%) | 6 ( 66.7%) | 12 ( 66.7%) |
| **Race** | American Indian or  Alaska Native | 1 ( 11.1%) | 0 ( 0.0%) | 1 ( 5.6%) |
|  | Asian | 0 ( 0.0%) | 0 ( 0.0%) | 0 ( 0.0%) |
|  | Black or African American | 1 ( 11.1%) | 0 ( 0.0%) | 1 ( 5.6%) |
|  | Native Hawaiian or  Other Pacific Islander | 0 ( 0.0%) | 0 ( 0.0%) | 0 ( 0.0%) |
|  | White | 7 ( 77.8%) | 9 (100.0%) | 16 ( 88.9%) |
| **Weight (kg)** | Mean (SD) | 80.18 (8.96) | 73.70 (13.21) | 76.94 (11.44) |
| **Height (cm)** | Mean (SD) | 178.17 (5.06) | 174.33 (6.88) | 176.25 (6.18) |
| **Body mass Index (kg/m^2^)** | Mean (SD) | 25.23 (2.27) | 24.15 (3.26) | 24.69 (2.78) |

Abbreviations: SAD= single-ascending dose, SD= standard deviation.
