# Supplementary material for: Safety, Tolerability, and Pharmacokinetics of Mevidalen (LY3154207), a Centrally Acting Dopamine D1 Receptor‐Positive Allosteric Modulator (D1PAM), in Healthy Subjects
Source: Clin Pharmacol Drug Dev. 2020 Oct 7;10(4):393–403. doi: 10.1002/cpdd.874 (PMC8048550; doi:10.1002/cpdd.874)
Supplement: Supplementary file 2 — Supplementary information [file CPDD-10-393-s002.doc]

**Table S1B. SAD-Subject Demographics and Other Baseline Characteristics**

| **Part B** | | | | | |
| --- | --- | --- | --- | --- | --- |
|  |  | Placebo | 25 mg | 75 mg | Overall |
| **Number of Subjects** |  | 8 | 8 | 8 | 24 |
| **Age (Years)** | Mean (SD) | 27.8 (5.4) | 30.4 (11.2) | 30.0 (12.5) | 29.4 (9.8) |
| **Sex** | Male | 7 ( 87.5%) | 7 ( 87.5%) | 8 (100.0%) | 22 ( 91.7%) |
|  | Female | 1 (12.5%) | 1 (12.5%) | 0 ( 0.0%) | 2 ( 8.3%) |
| **Ethnicity** | Hispanic or Latino | 1 ( 12.5%) | 0 ( 0.0%) | 1 ( 12.5%) | 2 ( 8.3%) |
|  | Not Hispanic or Latino | 7 ( 87.5%) | 8 (100.0%) | 7 ( 87.5%) | 22 ( 91.7%) |
| **Race** | American Indian or Alaska Native | 0 ( 0.0%) | 1 (12.5%) | 0 ( 0.0%) | 1 ( 4.2%) |
|  | Asian | 0 ( 0.0%) | 0 ( 0.0%) | 0 ( 0.0%) | 0 ( 0.0%) |
|  | Black or  African American | 1 (12.5%) | 0 ( 0.0%) | 0 ( 0.0%) | 1 ( 4.2%) |
|  | Native Hawaiian or  Other Pacific Islander | 0 ( 0.0%) | 0 ( 0.0%) | 0 ( 0.0%) | 0 ( 0.0%) |
|  | White | 7 ( 87.5%) | 7 ( 87.5%) | 8 (100.0%) | 22 ( 91.7%) |
| **Weight (kg)** | Mean (SD) | 75.24 (8.92) | 84.49 (18.88) | 76.91 (10.34) | 78.88 (13.50) |
| **Height (cm)** | Mean (SD) | 170.31 (4.76) | 180.94 (8.27) | 176.63 (7.74) | 176.63 (8.22) |
| **Body mass Index (kg/m^2^)** | Mean (SD) | 25.89 (2.34) | 25.73 (4.73) | 24.06 (2.54) | 25.22 (3.34) |

Abbreviations: SAD= single ascending dose, SD= standard deviation.
