# Supplementary material for: Safety, Tolerability, and Pharmacokinetics of Mevidalen (LY3154207), a Centrally Acting Dopamine D1 Receptor‐Positive Allosteric Modulator (D1PAM), in Healthy Subjects
Source: Clin Pharmacol Drug Dev. 2020 Oct 7;10(4):393–403. doi: 10.1002/cpdd.874 (PMC8048550; doi:10.1002/cpdd.874)
Supplement: Supplementary file 3 — Supplementary information [file CPDD-10-393-s001.doc]

**Table S2. MAD- Subject Demographics and Other Baseline Characteristics**

|  |  | **Placebo** | **15 mg** | **30 mg** | **75 mg** | **150 mg** | **Overall** |
| --- | --- | --- | --- | --- | --- | --- | --- |
| **Number of Subjects** |  | 12 | 9 | 9 | 9 | 9 | 48 |
| **Age (Years)** | Mean (SD) | 41.3 (9.8) | 36.9 (9.8) | 44.7 (13.3) | 39.1 (12.3) | 34.3 (12.6) | 39.4 (11.6) |
| **Sex** | Male | 10 (83.3%) | 9 (100.0%) | 8 (88.9%) | 7 (77.8%) | 9 (100.0%) | 43( 89.6%) |
|  | Female | 2 (16.7%) | 0 (0.0%) | 1 (11.1%) | 2 (22.2%) | 0 (0.0%) | 5 (10.4%) |
| **Ethnicity** | Hispanic or Latino | 1 ( 8.3%) | 0 (0.0%) | 1 (11.1%) | 3 (33.3%) | 0 (0.0%) | 5 (10.4%) |
|  | Not Hispanic or Latino | 11  ( 91.7%) | 9  (100.0%) | 8  ( 88.9%) | 6  (66.7%) | 9  (100.0%) | 43  ( 89.6%) |
| **Race** | American Indian or  Alaska Native | 0  (0.0%) | 0  (0.0%) | 0  (0.0%) | 0  (0.0%) | 0  (0.0%) | 0  (0.0%) |
|  | Asian | 4 (33.3%) | 3 (33.3%) | 3 (33.3%) | 2 (22.2%) | 3 (33.3%) | 15 (31.3%) |
|  | Black or  African American | 3  (25%) | 2  (22.2%) | 3  (33.3%) | 3  (33.3%) | 2  (22.2%) | 13  (27.1%) |
|  | Native Hawaiian or  Other Pacific Islander | 0  (0.0%) | 0  (0.0%) | 0  (0.0%) | 0  (0.0%) | 0  (0.0%) | 0  (0.0%) |
|  | White | 5 ( 41.7%) | 4 (44.4%) | 3 (33.3%) | 3 (33.3%) | 4 (44.4%) | 19 (36.9%) |
|  | Multiple | (0.0%) | 0 (0.0%) | 0 (0.0%) | 1 (11.1%) | 0 (0.0%) | 1 (2.1%) |
| **Population** | Japanese | 4 (33.3%) | 3 (33.3%) | 3 (33.3%) | 2 (22.2%) | 3 (33.3%) | 15 (31.3%) |
|  | Non-Japanese | 8 (66.7%) | 6 (66.7%) | 6 (66.7%) | 7 (77.8%) | 6 (66.7%) | 33 (68.8%) |
| **Weight (Kg)** | Mean (SD) | 75.49 (12.57) | 77.86 (13.43) | 74.31 (13.86) | 71.27 (13.43) | 75.64 (16.11) | 74.20 (13.44) |
| **Height (cm)** | Mean (SD) | 170.25 (8.82) | 178.94 (6.73) | 173.68 (9.30) | 172.89 (10.65) | 179.44 (7.25) | 174.74 (9.08) |
| **Body mass Index (Kg/m^2^)** | Mean (SD) | 25.87 (2.63) | 24.18 (2.94) | 24.44 (2.47) | 23.63 (2.48) | 23.35 (3.97) | 24.14 (2.86) |

Abbreviations: MAD= multiple-ascending dose, SD= standard deviation.
