# Supplementary material for: Safety, Tolerability, and Pharmacokinetics of Mevidalen (LY3154207), a Centrally Acting Dopamine D1 Receptor‐Positive Allosteric Modulator (D1PAM), in Healthy Subjects
Source: Clin Pharmacol Drug Dev. 2020 Oct 7;10(4):393–403. doi: 10.1002/cpdd.874 (PMC8048550; doi:10.1002/cpdd.874)
Supplement: Supplementary file 4 — Supplementary information [file CPDD-10-393-s003.doc]

**Supplementary Materials**

***Pharmacokinetic Assessments and bioanalytical method***

Concentrations of mevidalen in plasma, CSF, and urine from subjects receiving mevidalen were assayed using a validated liquid chromatography/tandem mass spectrometry method at Covance Laboratories Inc. (Madison, Wisconsin, USA). The lower limit of quantification (LLOQ) of mevidalen plasma was 0.500 ng/mL, and the upper limit of quantification was 2500 ng/mL. The LLOQ of mevidalen urine was 1.00 ng/mL, and the upper limit of quantification was 5000.00 ng/mL. The LLOQ of CSF was 0.500 ng/mL, and the upper limit of quantification was 2500.000 ng/mL. Internal standard(s): LSN3203139; MS type/conditions: Positive Ion Electrospray (ESI+) MRM Mode; Detection: mevidalen 450.2→246.1; LSN3203139 456.2→252.1. Mobile phase: A: 5 mM Ammonium Bicarbonate in Water [MA1];B: Acetonitrile; Column: Waters, X-Bridge, 30 x 2.1 mm, 5 μm particle size, 30°C; Flow rate: 1.5 mL/min; Sample preparation: The analytes were extracted from plasma by precipitation of proteins and analyzed using liquid chromatography (LC) with tandem mass spectrometric detection (MS/MS). Validation inter-assay accuracy and precision: mevidalen %RE: -2.2 to 7.8%; mevidalen %RSD: 2.0 to 6.9%. Validation intra-assay accuracy and precision: mevidalen %RE: -2.7 to 12.2%; mevidalen %RSD: 1.3 to 7.7%. All samples were analyzed in the timeframe supported by frozen stability storage data. Mevidalen was stable for up to 179 days when stored at approximately -70°C.
